# Genetic composition of facultatively anadromous brown trout *Salmo trutta* L. in a freshwater‐lake system

**DOI:** 10.1111/jfb.70553

**Published:** 2026-06-30

**Authors:** Ingerid J. Hagen, Rachel A. Paterson, Sten Karlsson, Ola H. Diserud, Geir H. Bolstad, Eva M. Ulvan, Ola Ugedal, Tor F. Næsje

**Affiliations:** ^1^ Norwegian Institute for Nature Research Trondheim Norway

**Keywords:** anadromy, genetic structure, potamodromy, sea trout, straying

## Abstract

Brown trout (*Salmo trutta*) has adapted to a wide range of habitats and displays a variety of life‐history strategies. Being facultatively anadromous, populations without migration barriers to the sea consist of both resident and anadromous individuals in different proportions. This affects genetic drift and the level of gene flow between populations, and therefore shapes the metapopulation structure. Here, utilizing genetic variation at 95 SNP markers, we describe the genetic structure and effective size of a brown trout population inhabiting the Fremstad catchment that supports both anadromous and freshwater resident life histories. Compared to other sea trout populations in the region, the Fremstad population is genetically divergent with a unique genetic signal. A weak and significant genetic structure was observed in the Fremstad catchment, and anadromous individuals were not assigned to a specific lake or stream. The effective population size of brown trout in the Fremstad catchment is large compared to the average annual entry of sea trout into the catchment; hence, a large proportion of non‐anadromous spawners likely contribute to the population. Approximately 2% of sea trout entering the catchment were strays from other populations. Because of a large proportion of non‐anadromous spawners, the proportion of strays into the overall spawning population is likely to be lower than observed among the sea trout, and contribute to the genetic divergence of the Fremstad population from other populations. This study adds to our understanding of the evolutionary drivers that affect the genetic structure of anadromous brown trout populations and demonstrates how small spawning streams can be evolutionarily important in supporting a large and genetically unique population.

## 1 INTRODUCTION

Brown trout (*Salmo trutta* L.) is a native salmonid of Eurasia and Northern Africa that has been successfully introduced to all continents except Antarctica (MacCrimmon & Marshall, 1968). Across its distribution, this species inhabits a wide spectrum of habitats, including rivers in temperate and sub‐tropical lowland regions as well as sub‐arctic and alpine catchments with prolonged seasonal snow cover. A high degree of variation in both morphological and life‐history traits contributes to this species' ability to successfully adapt to different environments. One such trait is anadromy, which is facultative in brown trout (Klemetsen et al., 2003) and can be adaptively advantageous as anadromous individuals (sea trout) foraging at sea typically attain higher body mass, and subsequently higher fecundity, than non‐anadromous individuals (Gross et al., 1988; Jensen et al., 2019; Jones et al., 2025; Jonsson, 1985). However, marine migrations are costly due to the energy‐demanding process of smoltification (Høgåsen, 1998) and because of the exposure to marine predators and parasites (Davidsen et al., 2017; Koed et al., 2006; Unger & Palm, 2016). In particular, increased exposure to the ectoparasite salmon lice (*Lepeophtheirus salmonis*) associated with the Atlantic salmon aquaculture industry is a key threat to sea trout survival and reproductive success (e.g., Fiske et al., 2024), and as a consequence, this parasite has the potential to alter the evolutionary trajectory of anadromy in brown trout (Bolstad et al., 2025).

Anadromy promotes connectivity between trout populations, and the movement of sea trout between watercourses can be considerable, with straying rates ranging from 3% to 15% in Norway (Berg & Berg, 1987; Jonsson & Jonsson, 2014) and more than 30% among Danish populations (Källo et al., 2022). However, movement is not necessarily congruent with gene flow, for which spawning and recruitment of offspring in a non‐natal river is required. Sea trout are known to transiently enter non‐natal rivers (Jensen et al., 2015), possibly due to predator avoidance (Thorstad et al., 2016) or overwintering (Jensen et al., 2015). While some individuals may migrate distances over 1000 km (King et al., 2024), most movement occurs between nearby watercourses less than 50 km away (Berg & Berg, 1987; Chat et al., 2022; Degerman et al., 2012; Thorstad et al., 2016).

Studies on the genetic structure of sea trout in Northern Europe based on nuclear genetic markers show that sea trout in different rivers are genetically distinct from each other (Bekkevold et al., 2020; Hagen et al., 2025; Hansen et al., 2007; Skaala, 1992; Skaala & Nævdal, 1989). Genetic structure can also develop in anadromous watersheds, as observed in anadromous tributaries to the river Luga, which drains into the Baltic Sea (Lehtonen et al., 2009), and the river Dart in southern England (Griffiths et al., 2009). Similarly, studies of large freshwater lake and river systems with adfluvial brown trout, such as the Inari Basin in Finland (Swatdipong et al., 2010), Lake Mjøsa in Norway (Linløkken et al., 2014) and Lake Pyaozero in Russia (Huusko et al., 2024), indicate limited admixture between different tributaries. These rivers and lake basins are large, and some sampling locations were up to 300 km apart (Swatdipong et al., 2010). However, genetic structure has also been observed in riverine brown trout from small non‐anadromous streams without apparent barriers to movement over distances spanning as small as 1–10 km (Andersson et al., 2022; Carlsson et al., 1999; Carlsson & Nilsson, 2000; Carlsson & Nilsson, 2001; Saha et al., 2022). These studies document the potential for brown trout to develop genetic structuring in the absence of migratory barriers, despite the documented propensity to move between watercourses. However, the genetic structure observed between Norwegian sea trout populations does not imply a simple relationship between genetic and geographic distance but instead shows that some populations are considerably more genetically divergent from their neighbouring populations at neutral nuclear loci than the mean genetic distance between the populations (Hagen et al., 2025; Hovgaard et al., 2006). The propensity for marine migration and duration of marine stays will be affected by freshwater foraging opportunities and will therefore vary between populations, according to characteristics of the local environment (Ferguson et al., 2019; Jensen et al., 2015; L'Abee‐Lund et al., 1989). Anadromous catchments with lakes may promote a variety of life‐history strategies, such as lake or river residency, lacustrine–adfluvial habitat use or anadromy (reviewed in Ferguson et al., 2019). Possibly, some anadromous watercourses that support both potamodromous and anadromous life histories will allow for non‐anadromous individuals to contribute substantially to the gene pool, thus supporting a high effective population size that will reduce the effect of gene flow from other sea trout populations.

**FIGURE 1:**
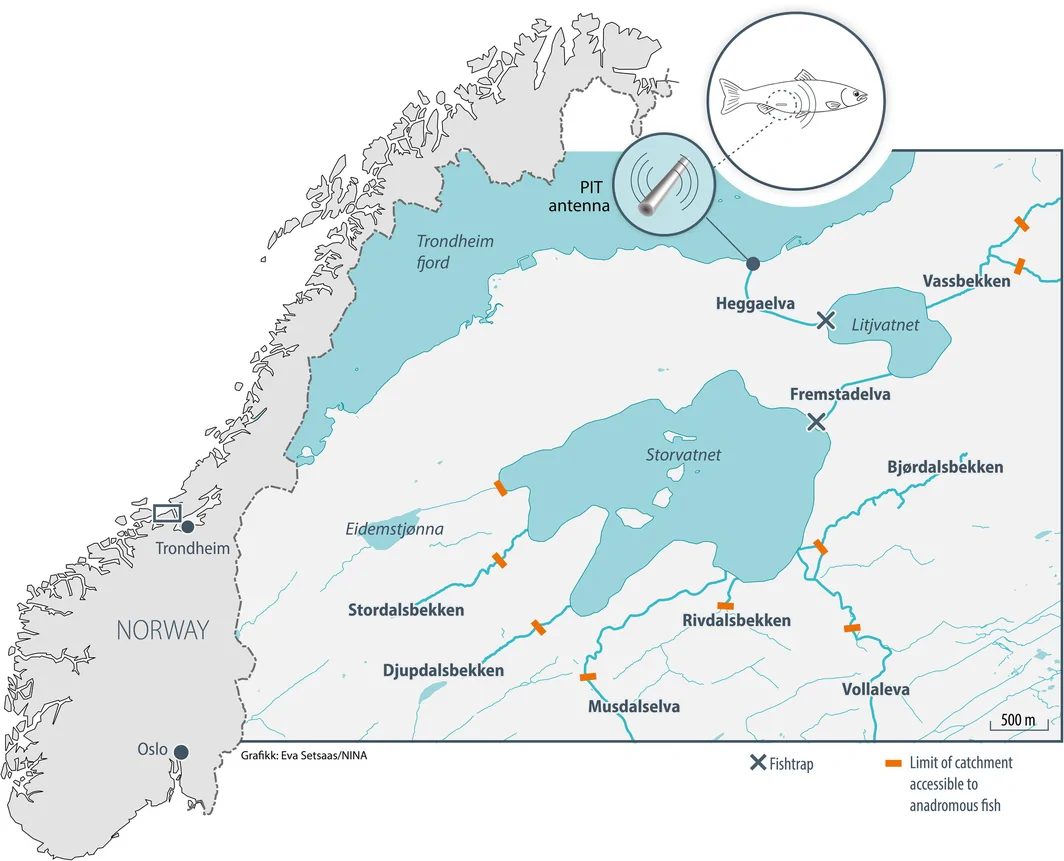
The Fremstad catchment, Trondheimsfjord, Norway. Orange bars indicate barriers to upstream migration. Crosses indicate fish traps. A passive integrated transponder (PIT) antenna is located at the marine outlet of the river Heggaelva. A two‐way trap is located in Heggaelva, and a one‐way trap is located at the inlet of the river Fremstadelva. Juveniles were sampled in all spawning creeks draining into the lakes (details in Table S1).

The focus of this study is on a watershed with lakes and rivers found to be genetically differentiated from other sea trout populations in the region (Hagen et al., 2025), the Fremstad catchment at the entrance of the Trondheimsfjord (Norway) (Figure 1). The complexity of the system allows for combinations of potamodromy and anadromy, and is therefore suitable for testing the hypothesis that the genetic divergence of the Fremstad catchment is driven by a large proportion of non‐anadromous spawners and a proportionally lower immigration rate. Moreover, sea trout in the Fremstad catchment are severely affected by salmon lice (Fiske et al., 2024) and could therefore be under an anthropogenically induced selection pressure that shifts the population away from anadromy (Bolstad et al., 2025). A first step towards understanding how salmon lice affects this important life‐history trait is to estimate the proportion of resident versus anadromous spawners. We sampled 1796 individuals from the Fremstad catchment, including up‐migrating and out‐migrating sea trout, fish from the two lakes and fish from streams running into the lakes. Trout from other rivers in the region were collected to be used as reference populations, and all individuals were genotyped for 96 nuclear single nucleotide polymorphisms (SNPs). We used these data to: (1) determine if trout in the Fremstad catchment can be considered a single population or whether there is considerable genetic structuring within the catchment, (2) discern if individuals that migrate to sea are a random subset of brown trout in the lakes and streams, (3) verify the previously suggested genetic divergence of the Fremstad catchment using an extended baseline of samples and populations, (4) determine the number of strays entering the Fremstad catchment and (5) estimate the effective size of the spawning population and relate the estimate to the proportion of anadromous and resident fish in the population. We discuss putative biological mechanisms affecting the observed genetic structure and consider the implications for sea trout conservation management.

## 2 METHODS

### 2.1 The study system

The Fremstad catchment (area 27.6 km^2^, drainage 34.2 million m^3^ years^−1^; 63°370 N, 9°380E) is located at the entrance of Trondheimsfjord (Trøndelag, Norway), with approximately 8 km of the lower catchment being accessible to anadromous fish (Figure 1). The lower catchment consists of two lakes, Storvatnet [2.92 km^2^, max. depth 16 m, 6 m above sea level (masl)] and Litjvatnet (0.47 km^2^, max. depth 3 m, 5 masl), which are connected by the river Fremstadelva (0.8 km). Six lowland streams flow into Storvatnet, whereas the stream Vassbekken and river Fremstadelva (from Storvatnet) flow into Litjvatnet, and the river Heggaelva (1 km) connects Litjvatnet to the sea. The spawning habitat in the rivers Heggaelva and Fremstadelva is limited due to large proportions of these rivers being channelized (83 and 100% channelized, respectively; NVE 2024), and thus these rivers mainly serve to connect the catchment to the sea. The remaining seven streams draining into the lakes provide spawning habitat for trout, but are to varying degrees impacted by agriculture (0%–50% land use). Thus, this study system invites a potentially large variety of migration‐spawning life‐history strategies, including lacustrine–adfluvial, river residency, allacustrine, fluvial–adfluvial and anadromy. Current knowledge suggests that brown trout do not use the lakes for spawning. Upstream of Storvatnet is Langvatnet (0.06 km^2^, 161 masl), the only non‐anadromous lake in the Fremstad catchment harbouring a population of resident trout. A~15 m waterfall in the mid‐reaches of the spawning stream Musdalselva enables unidirectional downstream gene flow from Langvatnet to Storvatnet to occur, but is likely to be limited.

### 2.2 Field sampling

The spawning streams connected to lakes Storvatnet and Litjvatnet were surveyed for juvenile trout during June 2022 and November 2023, except for Djupdalsbekken, which was surveyed in June 2022 only. One or two reaches were surveyed in each stream by electrofishing, depending on the size of the stream and 34–60 juvenile trout were collected from each reach in the seven spawning streams (details in Supporting Information Table S1, Figure S1). Fish were euthanized using an overdose of benzocaine and preserved in 96% ethanol (2022) or frozen (2023). Trout from the lakes Storvatnet (*N* = 196) and Litjvatnet (*N* = 100) were sampled in June and September 2021 using Nordic multi‐panelled gill nets (30 × 1.5 m; mesh sizes 5, 6.25, 8, 10, 12.5, 15.5, 19.5, 24, 29, 35, 43, 55 mm) and SNSF floating gill nets (52 × 6 m, mesh sizes: 10, 12.5, 16, 19.5, 24, 30, 35, 45 mm), killed with a blow to the head and frozen until processed. In the laboratory, stream and lake fish were defrosted before being measured for total length (mm) (Figure S1), and a small tissue sample was obtained from the pectoral fin and fixed in 96% ethanol. Scale samples were collected from all individuals. The gender and reproductive status of the lake fish were determined by visually inspecting the gonads. The migratory life history of the individuals from Storvatnet whose gonads showed that spawning had occurred during previous seasons or that spawning would have occurred the coming season was assessed by scale reading to determine whether these individuals had been residential their whole life, or if marine migration had occurred (Elliott, 1996). Of the 37 individuals identified as either mature spawners or those that had previously spawned, three were found to have had at least one marine migration. This means that of the 196 individuals from Storvatnet, 17% were spawners with a non‐migratory life history. The mean weight of the non‐migratory spawners was 340 g, while the mean weight of immature individuals captured in Storvatnet was 142 g.

Trout out‐migrating from Storvatnet were captured at the outlet of Fremstadelva during Spring 2017, 2020 and 2021, and were fitted with a passive integrated transponder (PIT) tag to enable sea trout to be detected at the Heggaelva PIT antenna as they entered the marine environment (Figure 1, Figure S1; see Paterson et al., 2021 for detailed methods). Genetic material from out‐migrating sea trout was obtained by fixing the sterile needle used to tag each fish in ATL Buffer (Qiagen, Germany).

Sea trout up‐migrating from the Trondheimsfjord into the Fremstad catchment were captured in a fish trap in the upper reaches of the river Heggaelva during June–October 2023 (Figure 1). The trap was designed to catch all up‐migrating fish when operational and was operated three consecutive nights per week, hence approximately 43% of the sea trout that entered the catchment would have been caught. In total, 189 sea trout were captured during the 2023 season, and according to the proportion of time the trap was operated, the number of sea trout that entered the catchment was likely no more than ~450, not accounting for harvest (which is very limited) and predation due to American mink and Eurasian otter. Sea trout in the Fremstad catchment spawn in October, and due to decreasing temperatures at sea and in freshwater, migration to freshwater (both homing and overwintering in non‐natal freshwater habitat) is generally completed by the end of October (Paterson et al., 2021; Thorstad et al., 2016). Prior to release, captured fish were measured (length in mm, weight in g), a scale sample was carefully collected from each individual for DNA extraction, and any already present PIT tags were recorded. The mean weight of the up‐migratory individuals was 612 g. The gonadal state of these individuals was not checked, but the body size of these individuals allows for the assumption that they were likely mature and would spawn during the 2023 season. From scale reading, the age of the fish ranged between 2 and 9 years, hence they were produced during brood years 2011–2020. Smolt age ranged between 2 and 7 years, and the number of previous sea migrations ranged from 0 to 5. The trap was operational also in 2024 and 2025. In 2024, the trap was operational every day between June 6 and October (127‐day period), and 253 sea trout were caught. In 2025, the trap was operational every day between June 13 and October 31, except for 15 days due to high water levels, and during the 127 days, the trap caught 262 sea trout. The captures in the Heggaelva trap are regarded as providing a reliable approximation of the annual number of up‐migratory sea trout.

### 2.3 Ethics statement

The care and use of experimental animals complied with Norwegian Food Safety Authority animal welfare laws, guidelines and policies as approved by the Authority (permit numbers 11,313, 22,993, 29,144, 8631, 14,683, 22,993, 29,334).

### 2.4 Age distribution of sampled juveniles

The total length of juvenile trout collected from each spawning stream in 2022 and 2023 was visually inspected to identify size/age classes. The age of all individuals in the smallest and largest size classes, and a random sub‐sample of 15 individuals from the intermediate size class, was confirmed by counting the annuli from scale samples according to Elliott and Chambers (1996), and was used for estimating the age of all fish in the intermediate size class. Because sampling occurred in June 2022 and November 2023, the two sampling years were treated separately.

### 2.5 DNA extraction and genotyping

DNA was extracted from the scales, fin clips and PIT needles using DNEASY tissue kit (QIAGEN). We used a panel of 96 nuclear SNPs that were found to be variable in Danish brown trout (Bekkevold et al., 2020), are unlinked and evenly distributed on all 40 brown trout chromosomes (Saha et al., 2022). This panel of widely used 96 SNPs has proven representative for neutral variation in Scandinavian brown trout (Andersson et al., 2022; Hagen et al., 2025; Saha et al., 2022), and can also identify individuals of the sympatric species Atlantic salmon (*Salmo salar*), and hybrids between brown trout and Atlantic salmon. Samples were genotyped using the EP1 96.96 Dynamic array IFCs genotyping platform (Fluidigm). Samples with genotype success below 80% (*N* = 78) were removed, as were individuals identified as Atlantic salmon‐brown trout hybrids (*N* = 12), leaving 1706 samples from the Fremstad catchment for further analyses.

### 2.6 Statistical methods

To ensure high genetic data quality for the two large data sets used (within the Fremstad catchment and Fremstad together with 15 other populations in mid‐Norway), we investigated deviations from Hardy–Weinberg expectations using Genalex (Peakall & Smouse, 2006). According to an *α*‐level of 0.05, we used a Bonferroni correction (Bland & Altman, 1995) to adjust the probability values for significant deviation from Hardy–Weinberg expectations for each marker and sampling location. Within the Fremstad catchment, nine sampling locations were defined (seven streams and two lakes; leading to 860 separate tests), and in the data covering mid‐Norway, 16 sampling locations were defined (leading to 1440 separate tests). Following this procedure, one marker was removed from each of the data sets, leaving 95 markers in each data set.

Juvenile brown trout (particularly young of the year and one‐year‐olds) occupy suitable habitat near the redd from which they hatched, thus juveniles sampled in geographical proximity of each other may be closely related. Because strong family structure in the samples can inflate the observed F_ST_ (Allendorf & Phelps, 1981; Hansen et al., 1997; Wenburg et al., 1998), we identified full‐sibling groups with probabilities (inclusive and exclusive) of 0.9 or higher using the program COLONY (Jones & Wang, 2010) and removed all but one representative of each full‐sibling family group prior to downstream analyses by keeping the individual with the highest genotype rate. In cases where genotype rates were similar, the selection was random. COLONY was run with the possibility for polygamy for both males and females, without updating allele frequencies, without ‘sibship scaling’ and without ‘sibship prior’. The expected genotype error rate was set to a conservatively high estimate of 0.001. Within each stream, samples assumed to originate from the same brood year were analysed together, regardless of sampling year. As most sampled individuals were young‐of‐the‐year and one‐year‐olds, and sampling occurred during the two consecutive years 2022 and 2023, analysis in COLONY focused on the brood years 2020–2022. More family groups were observed for the 2020 brood year than for the 2022 brood year, probably due to the distribution of related individuals in the streams (Supporting Information Table S2). The time of year when field work was conducted (June 2022 and November 2023) would have contributed to this difference. In total, 118 individuals were removed, and these were not included in the downstream analyses.

To quantify potential temporal and spatial genetic structure within each stream, we calculated pairwise F_ST_ (Weir & Cockerham, 1984) with the package Hierfstat (Goudet, 2005) implemented in R (R Core Team, 2025) with groups defined by both reach within stream and sampling year. We used 1000 bootstraps across loci to estimate upper and lower 95% confidence limits for each pairwise F_ST_ value. Given that the 95% confidence intervals (CIs) do not overlap with zero, the estimate can be regarded as significantly different from zero at an *α*‐level of 0.05. The outcome of this analysis (Supporting Information Table S3) showed low and non‐significant pairwise values (not different from zero) for all within‐stream temporal and spatial combinations except Reach 1 and 2 in Vollaelva 2023 (F_ST_ = 0.007, 95% CI: 0.005–0.014), Reach 2 in 2022 and Reach 1 in 2023 (F_ST_ = 0.008, 95% CI: 0.001–0.014) and Bjørdalsbekken 2022 and 2023 (F_ST_ = 0.007, 95% CI: 0.001–0.014). Vollaelva is the largest spawning stream, and within Vollaelva, there was no consistent pattern as the other pairwise F_ST_‐values were not significantly different from zero. This means that Bjørdalsbekken was the only spawning stream with inter‐annual genetic difference. Because inter‐annual F_ST_ in Bjørdalsbekken was low with the lower 95% CI just above zero, and the other inter‐annual F_ST_‐values were inconsistent (Vollaelva) or non‐significant, samples from each stream were subsequently analysed as representatives of the respective stream in all following analyses. Similar pairwise F_ST_ analysis was conducted for the group of out‐migrating sea trout to detect potential genetic structure between sampling years (2017, 2020 and 2021), for which the lower 95% CI of all pairwise F_ST‐_values overlapped with zero, hence there was no significant genetic structure within the group of out‐migrating sea trout.

Pairwise genetic distances (F_ST_) were calculated between each stream, the two lakes and the out‐migrating sea trout. This analysis was based on the expectation that genetic structure would be observed between spawning streams due to the previously described propensity of brown trout to be structured into genetically distinct sub‐populations despite the lack of apparent migratory barriers. Moreover, we expect that brown trout in the spawning streams will have small genetic distances to brown trout captured in the lake that the respective streams drain into, and where brown trout from all the different streams reside. Lastly, genetically distinct sub‐populations in the different spawning streams in relation to the migrating sea trout will inform the contribution of the respective streams to sea trout produced in the Fremstad catchment.

Previous research has shown that the Fremstad catchment is genetically differentiated from geographically close populations, with a mean pairwise F_ST_ of 0.104 against the other populations, versus a mean pairwise F_ST_ of 0.056 between other populations (excluding Fremstad), based on the same genetic markers as used in this study (Hagen et al., 2025). To further examine this genetic divergence of the Fremstad catchment, we sampled juvenile trout from 10 additional rivers in the region. We calculated pairwise F_ST_ as described above using the previously published genetic data (five populations; 180 individuals in total), the data from the 10 additional reference populations (652 individuals in total) and the 366 out‐migrating sea trout from the Fremstad catchment. The genetic distance between the respective populations was visualized using a principal coordinate analysis implemented in the R package PopGenReport (Adamack & Gruber, 2014).

The genetic distance of the Fremstad Sea trout population to the other populations (Hagen et al., 2025) allows for the identification of sea trout originating from the Fremstad catchment. We therefore conducted genetic assignment of the sea trout captured in the Heggaelva fish trap as they entered the catchment from the sea. The identification of individuals genetically assigned to other populations provides an estimate of the rate of straying sea trout into the Fremstad catchment. Nine individuals were removed due to poor genotype rate, and the remaining 180 individuals were assigned a population of origin using the DAPC method in the R package Adegenet (Jombart, 2008) and reference data from the populations described above. Using DAPC, two clusters were assumed to be most likely (Supporting Information Figure S2) due to the substantial divergence between Fremstad and the other 15 populations.

The effective population size (N_e_) is generally smaller than the number of adults (N_c_), as individuals usually differ in their reproductive success (Wright, 1931). For salmonids, the following N_e_/N_c_ ratios have been reported: 0.2 for Atlantic salmon (Hindar et al., 2004), <0.2 for chinook salmon *Oncorhynchus tshawytscha* (Shrimpton & Heath, 2003), <0.7 for steelhead trout, *Oncorhynchus mykiss* (Ardren & Kapuscinski, 2003) and <0.2 for brown trout (Palm et al., 2003). An observed N_e_/N_c_ ratio that greatly exceeds the previously observed ratios for salmonids, as well as the general expectation that N_e_/N_c_ <1, would indicate that more spawners than the observed sea trout contribute to the population. Brown trout are iteroparous, and anadromous populations are subjected to varying degrees of gene flow, thus violating two of the underlying assumptions for estimating N_e_ (Waples, 2006). However, if the sample from the population includes a number of cohorts that is equal to the generation length of the population, then the N_e_ estimate will represent the N_e_ for a generation (Waples & Do, 2008), and if the sample represents only individuals produced in one season, the N_e_ estimate represents the effective number of breeders (spawners) that season (N_eB_) (Waples & Do, 2008), while gene flow will inflate the estimate (Serbezov et al., 2012). We estimated the effective population size (N_e_) in Fremstad using three different sample sets: (1) The returning adults, which included all freshwater‐ and sea ages and therefore represents a whole generation (or more) of sea trout (*N* = 176); (2) The PIT‐tagged out‐migratory sea trout that was captured and PIT marked in Storvatnet in 2017, 2020 and 2021 (*N* = 364; two individuals removed after DAPC analysis). These individuals do not represent the contribution by spawners in Vassbekken; (3) The trout captured in the two lakes represent the production in all streams (*N* = 285). Individuals from Storvatnet were aged (mean age = 3.84 years, mean length = 224 mm) (Ekren, 2023). The PIT‐marked individuals also originated in Storvatnet, and their size distribution indicates that these individuals represent age groups from 1 year and up to 5 years or more (Ekren, 2023). This means that the latter data sets include the production from several brood years, possibly 8–10 cohorts. The effective population size was estimated using the linkage disequilibrium method, as implemented in the program LDNe (Waples & Do, 2008). For this purpose, individuals diagnosed as certain strays following genetic assignment (described above) were excluded from the analysis, as these probably did not originate in the Fremstad catchment. Moreover, the effective number of breeders (N_eB_) was estimated for each brood year that was represented by the samples from the spawning streams. The estimates of N_eB_ were generated from the aforementioned sibship analysis in COLONY (Wang, 2009) for the brood years 2020, 2021 and 2022. Because not all spawning streams were represented in each brood year, and some family groups were probably not represented in the sampling, all estimates of N_eB_ are biased downwards.

**FIGURE 2:**
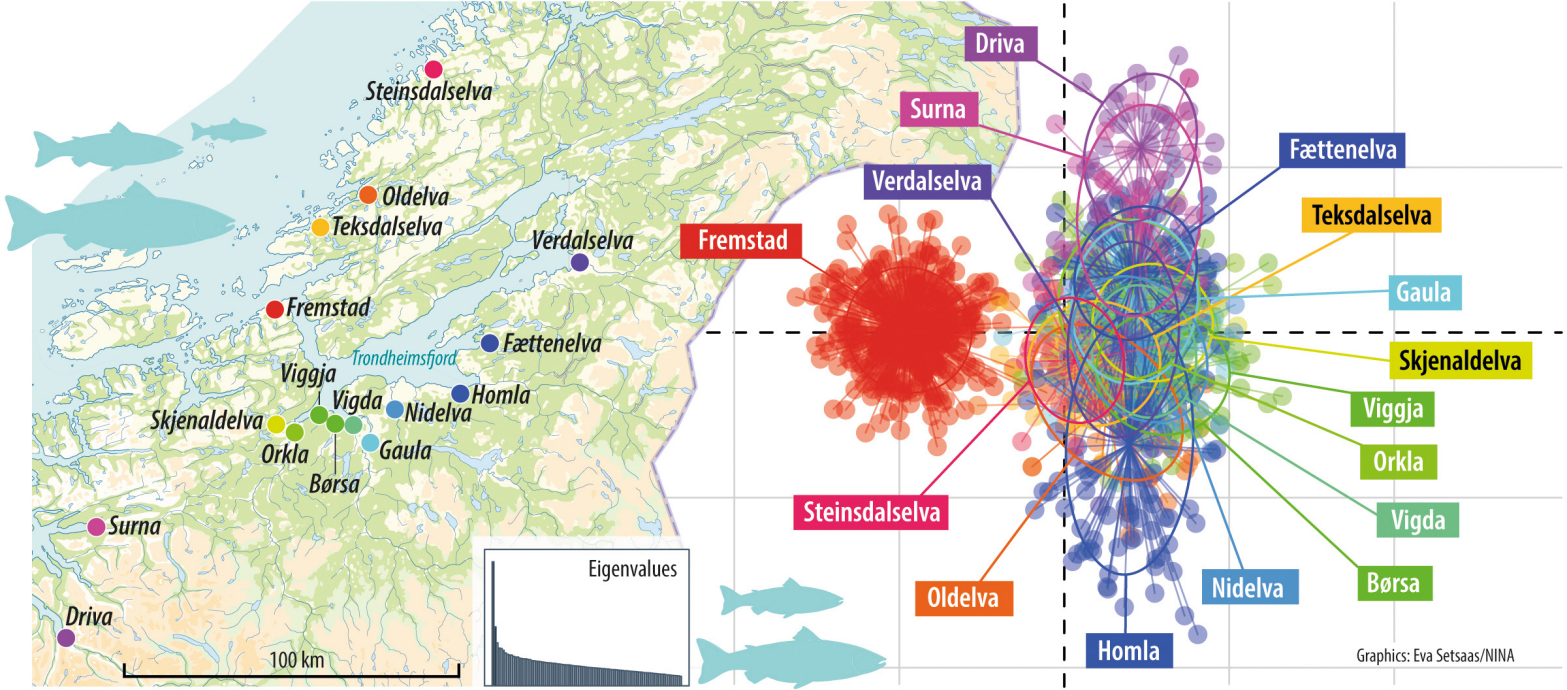
The geographic location of the Fremstad catchment and 15 other sea trout populations in mid‐Norway, together with a principal coordinate plot (PCoA) visualizing the genetic diversity among populations and sampled individuals. The eigenvalues bar plot visualizes the amount of variation explained by each principal component, out of which the first two are used in the PCoA plot. For details on genetic distances between populations, see Table S4. Eigenvalues are illustrated in Figure S3.

In addition to evaluating the ratio between effective population size and the number of anadromous trout in the Fremstad catchment, we also compared and contrasted this estimate with a different river in the region (Vigda; Figure 2), which allows almost exclusively an anadromous life history. In Vigda, adult annual numbers of spawners have been estimated for the years 2019–2025 (https://bestand.nina.no/#/122.2Z), and genetic samples of 214 PIT‐marked out‐migratory smolt in 2016, 2017, 2018 and 2022 have been collected, representing around nine cohorts. This sample was used for estimating the effective population size in LDNe, as described above. Vigda is ~13 km long, and the available spawning habitat in Vigda is much larger than the available habitat in Fremstad (95,014 m^2^ in Vigda vs 22,357 m^2^ in Fremstad), albeit in Vigda, the habitat is also utilized by Atlantic salmon. The anadromous habitat in Vigda is riverine, without anadromous lakes. The annual number of up‐migratory sea trout in Vigda is similar (>215 sea trout entered annually during 2019–2021, which are the years most relevant to the N_e_ estimate) to Fremstad (approximately 250–450 sea trout enter annually). From the average annual number of up‐migrating anadromous trout and the available spawning area, without considering the contribution of non‐anadromous brown trout, we expect Vigda to support a similar effective population size as Fremstad. Underlying this expectation is the assumption that the numbers of sea trout entering the two catchments have not changed considerably from the run years that the N_e_ estimates are based on, and the later years from which census data exist. Populations of anadromous salmonids in Norway are closely monitored by management authorities, and there is no information suggesting that population sizes of sea trout in these two systems have changed notably over the time period covered by this study.

## 3 RESULTS

### 3.1 Genetic structure within the Fremstad catchment

The observed genetic structure was consistent with the geography of the catchment: Streams that were geographically close to each other had lower pairwise F_ST_ values, and the two lakes had low pairwise F_ST_ values towards spawning streams that drain into each respective lake (Table 1). The genetic distances (F_ST_) between the group of out‐migrating sea trout and trout collected in the two lakes were low (F_ST_ = 0.002 for Storvatnet and F_ST_ = 0.007 for Litjvatnet). Similarly, low differentiation was observed between the out‐migrating sea trout and juveniles from the spawning streams draining into Storvatnet (mean pairwise F_ST_ = 0.005), and particularly towards Bjørdalsbekken, for which the 95% CI for the respective pairwise F_ST_ value overlapped with zero and was therefore not significantly different from zero. The highest pairwise F_ST_ between the out‐migrating sea trout group and any spawning stream was towards Vassbekken (F_ST_ = 0.013), which is expected, as the individuals in the out‐migrating sea trout group were caught in and tagged in Storvatnet, while Vassbekken drains into Litjvatnet, and probably did not contribute much to the out‐migrating sea trout that resided in Storvatnet prior to migration. Vassbekken may give rise to sea trout that reside in Litjvatnet prior to migration, but this pattern cannot be discerned from the current data. The weak and non‐significant genetic differences observed between spawning streams that drain into Storvatnet and the group of out‐migrating sea trout suggest that all these spawning streams contribute to the sea‐trout group. While the observed genetic structure within the catchment is weak, it reflects the geography of the catchment and is therefore likely to be biologically meaningful. However, the average pairwise F_ST_ for the catchment (0.008) is much lower than the pairwise F_ST_ values observed between catchments in the Trondheimsfjord (see the results below), thus indicating more gene flow within the Fremstad catchment than between catchments.

**TABLE 1:** Pairwise F_ST_ values (below diagonal) for samples collected in the Fremstad catchment.

	Spawning streams							Lakes		Migrating sea trout
	Stordals‐bekken	Djupdals‐bekken	Musdals‐elva	Rivdals‐bekken	Vollaelva	Bjørdals‐bekken	Vass‐bekken	Stor‐vatnet	Litj‐vatnet	
Stordalsbekken	–	0.003 0.011	0.002 0.011	0.003 0.014	0.004 0.011	0.002 0.009	0.011 0.025	0.001 0.006	0.007 0.018	0.003 0.007
Djupdalsbekken	0.007	–	0.004 0.015	0.003 0.015	0.006 0.015	0.003 0.011	0.008 0.020	0.003 0.010	0.008 0.018	0.004 0.013
Musdalselva	0.005	0.009	–	0.003 0.016	0.006 0.015	0.003 0.014	0.013 0.028	0.001 0.011	0.010 0.024	0.003 0.012
Rivdalsbekken	0.008	0.008	0.008	–	0.004 0.014	0.004 0.013	0.009 0.021	0.002 0.011	0.006 0.016	0.003 0.013
Vollaelva	0.008	**0.010**	**0.010**	0.008	–	*0.000* *0.005*	0.008 0.016	0.002 0.004	0.005 0.011	0.002 0.005
Bjørdalsbekken	0.005	0.007	0.008	0.007	*0.003*	–	0.006 0.012	*0.000* *0.003*	0.002 0.007	*0.000* *0.003*
Vassbekken	**0.017**	**0.014**	**0.020**	**0.014**	**0.012**	0.009	–	0.007 0.015	*0.000* *0.005*	0.008 0.018
Storvatnet	0.003	0.006	0.005	0.006	0.003	*0.002*	**0.011**	–	0.004 0.011	0.001 0.003
Litjvatnet	**0.012**	**0.013**	**0.017**	**0.011**	0.008	0.004	*0.002*	0.007	–	0.003 0.011
Migrating sea trout	0.005	0.009	0.006	0.007	0.004	*0.001*	**0.013**	0.002	0.007	‐

*Note*: Above the diagonal are 95% confidence intervals for the respective F_ST_ values. The different locations/groups comprise seven spawning streams, two lakes and anadromous trout known to have migrated to the sea (migrating sea trout). F_ST_ values ≥0.01 are indicated with bold font, and non‐significant F_ST_ values are indicated with italics.

### 3.2 Genetic structure among catchments

Increasing the number of individuals to represent the Fremstad catchment by more than 10‐fold and increasing the number of sampled populations in mid‐Norway from five to 15 led to a mean pairwise F_ST_ value for the Fremstad sea trout population against all other populations of 0.100, which is similar to the previously reported value in Hagen et al. (2025), thus verifying the genetic divergence of the Fremstad population. In comparison, the mean F_ST_ based on all population pairs, excluding Fremstad, was 0.039 (Figure 2, Table S4, Figure S3). A principal coordinate plot (PCoA) of Fremstad and 15 other sea trout populations in mid‐Norway illustrates the genetic diversity among sampled individuals in the Fremstad catchment, as well as the genetic divergence of the Fremstad population from other populations. The Fremstad Sea trout population is represented by a much larger number of individuals than the other populations (366 in Fremstad vs. on average 55 from each of the others). The tight cluster formed by the Fremstad individuals illustrates that this population is genetically uniform and unique (Figure 2). Moreover, while the populations of Driva and Steinsdalselva are separated by a greater geographic distance (swimming distance: 193 km), they are less genetically divergent from each other than Fremstad is from any other populations in the region, including Vigda and Gaula, which are located 50 and 52 km away, respectively.

### 3.3 Genetic identification of immigrants

The trap in Heggaelva was designed to catch individuals that migrated into the catchment from the sea, and individuals caught in the trap were therefore sea trout that entered freshwater after foraging in the marine environment. Of the 189 individuals that entered the trap, 180 were successfully genotyped. Genetic assignment of these 180 individuals showed that 176 sea trout (97.8%) were assigned to the Fremstad population with a probability >0.995. Four individuals (2.2%) were assigned to the cluster representing other populations, with a probability of 0.99. These data suggest that 2.2% of the sea trout that entered the Fremstad catchment originated from other populations. Henceforth, these four individuals are referred to as strays. Of the Fremstad Sea trout reference samples (366 out‐migrating sea trout), two individuals (0.006%) were assigned to the non‐Fremstad cluster with a mean probability of 0.99. Six individuals (0.008%) from other reference populations were assigned to the Fremstad population with a mean probability of 0.86. The four strays were immigrants into the Fremstad catchment; however, as their reproductive success is not known, we cannot extrapolate a rate of gene flow from these individuals.

### 3.4 Effective size (N_e_) of the Fremstad population and the annual number of sea trout

The effective population size (N_e_) in Fremstad was estimated from three different data sets. Sea trout homing to the Fremstad catchment in 2023 gave an N_e_ of 615 (95% CI = 382–1409). Out‐migratory sea trout gave an N_e_ of 623 (95% CI = 482–857), and the individuals residing in the lakes gave an N_e_ of 727 (95% CI = 510–1206). All three approaches suggest that N_e_ within the Fremstad catchment is around 600–700. For the brood years 2020, 2021 and 2022, the effective number of breeders (N_eB_) was estimated and found to be 272 in 2020 (six streams sampled), 233 in 2021 (four streams sampled) and 441 in 2022 (six streams sampled). The fact that not all streams were sampled means that N_eB_ was underestimated for all years. The brood year 2020 is most likely severely underestimated because sampling covering this brood year was conducted in June, and many of the collected individuals (mostly one‐year‐olds) were full‐sibs (see Supporting Information Table S2), hence the trout in the streams had not yet moved away from the area around the redd where they hatched. Also, not the entire lengths of each stream were surveyed, and sampling would therefore have missed an unknown number of family groups. Consequently, a lower proportion of contributing parents would be represented among the collected offspring from the 2020 brood year than for the 2022 brood year, which was mainly covered by sampling in November 2023. Only four out of seven streams were represented in the 2021 brood year, while six streams were represented in the 2022 brood year sample and therefore produced the most reliable estimate of N_eB_ for Fremstad. However, the estimate for the 2022 brood year did not include Djupdalsbekken (N_eB_ of 24 and 58 for 2020 and 2021, respectively), and some parents were likely not represented among the collected offspring, meaning that the N_eB_ of 441 must be regarded as the lowest possible effective number of parents this year. The number of sea trout that entered the catchment in 2022 is not known, as the trap was not operational this year. However, in 2023, at least 189 but probably not more than ~450 sea trout entered the catchment, 253 sea trout up‐migrated in 2024, and at least 263 sea trout up‐migrated in 2025. For 2025, the number may be slightly higher due to 15 days during which the trap was not operational. In the likely scenario that the number of sea trout in 2022 was comparable to the later years, the effective number of breeders this year exceeded the number of sea trout that entered the catchment.

For the ecological comparison, Vigda, the effective population size was estimated to be 145 (95% CI = 126–167), which is considerably lower than any of the estimates for Fremstad, despite Vigda having more available spawning habitat and similar annual numbers of sea trout entering the river. The proportion between effective population size and approximate annual entry of sea trout is much lower in Vigda than in Fremstad, which adds support to the hypothesis that more breeders than sea trout contribute to reproduction in Fremstad, elevating the effective population size. Observations of 34 non‐anadromous individuals (freshwater life history confirmed by scale reading) that either had spawned previously or had mature gonads and would therefore spawn the coming season, among the individuals collected in Storvatnet (details in Methods), confirms that spawning without prior marine migration is common in Fremstad.

## 4 DISCUSSION

In this study, we have investigated the genetic structure of brown trout in an anadromous catchment that allows for both anadromous and potamodromous life‐history strategies. Within the Fremstad catchment, we found a weak but biologically meaningful genetic structure, which reflected the geography of the catchment. The weak genetic structuring in the Fremstad catchment suggests considerable gene flow between streams, while the genetic distances between catchments are much larger. Moreover, while the data at hand cannot inform on the contribution by different spawning streams to the production of sea trout in the Fremstad catchment, slight genetic differentiation of the out‐migratory sea trout from individuals in spawning streams and lakes suggests that the sea trout belong to the same gene pool as the juveniles produced in the different streams, as well as the lake‐residing individuals. Based on the two aforementioned observations, we conclude that trout in the Fremstad catchment can be managed as one population, and that anadromous brown trout have their origin in all spawning streams. Albeit biologically meaningful in terms of geography, it is possible that the weak genetic structuring observed based on samples of juvenile fish may have been inflated by genetic drift due to relatively few effective breeders represented among the collected samples, and that a large proportion of the spawners reproduce once or more before migrating to sea and may therefore be prone to spawn geographically close to where they hatched. In comparison, considerable genetic structuring (F_ST_ >0.04 based on the same marker panel as used here) was found in brown trout inhabiting rivers draining into Selbusjøen, a landlocked lake and river system in central Norway (Wacker et al., 2025). In a small Swedish lake, large genetic differences (F_ST_ of 0.24 based on the same SNPs as used here) between sympatric demes of brown trout were found (Saha et al., 2022), confirming the allozyme‐based observation by Ryman et al. (1979), while Andersson et al. (2022) reported large genetic differences between isolated Swedish brown trout populations but also clear genetic differences within catchments. Genetic differences (F_ST_ of 0.04 or higher based on the same marker panel as used here) were also found in brown trout collected in rivers draining into the lake Suldalsvatnet in southern Norway (Karlsson et al., 2025). The latter lake and river system is not landlocked, but access to Suldalsvatnet by anadromous individuals is restricted, and the contribution by anadromous spawners is therefore low. Hindar et al. (1991) studied trout in landlocked and anadromous reaches of the Vosso catchment in southern Norway and found no or small genetic differences between resident and anadromous trout in anadromous reaches but strong genetic separation between trout above migration barriers. The lack of genetic structuring in anadromous reaches reported by Hindar et al. (1991) mirrors the current observations within Fremstad, and it is possible that the presence of sea trout in the Fremstad system is homogenizing the gene pool more than would otherwise be the case in a non‐anadromous catchment.

In this study, we have confirmed the striking genetic divergence of the Fremstad Sea trout population compared to other nearby populations, as was previously observed in Hagen et al. (2025). Our results support the hypothesis that the genetic divergence is associated with a large effective population size elevated by non‐anadromous spawners and, subsequently, also a low rate of gene flow into the Fremstad catchment. It is possible that complex lake and river systems like the Fremstad catchment are ecologically different from the habitat in anadromous systems that are entirely riverine. Different ecologies will likely lead to different selection pressures, which again may negatively affect the reproductive success of strays entering the system. However, the detection of genomic regions under selection requires high‐density genome‐wide tools, and the marker set used here does not suffice. Our results indicate that there is a large proportion of non‐anadromous spawners in the Fremstad catchment, and these spawners make a considerable contribution to the overall gene pool; hence, the proportion of strays in the spawning population will be lower than the 2.2% observed among the sea trout. The large effective population size makes the gene pool less susceptible to genetic drift, which again is expected to increase the efficiency of natural selection and local adaptation (Walsh & Lynch, 2018). Hagen et al. (2025) showed that the large Norwegian fjords to some degree define a metapopulation structure, but that genetic distance between Norwegian sea trout populations varies greatly and does not follow a simple isolation by the distance pattern (Hagen et al., 2025; Hovgaard et al., 2006). This study presents a mechanism that advances our understanding of the evolutionary drivers shaping the genetic structure of sea trout.

The following observations indicate that the spawning population in Fremstad consists of non‐migratory individuals in addition to the sea trout: The effective number of breeders could be estimated (albeit with a downward bias) for the brood year 2022, and indicated that the number of parents that contributed to the production of the observed offspring was probably higher than the likely number of sea trout in the catchment. Also, we found that the effective size of the Fremstad trout population was considerably higher (N_e_ = 600–700) than the nearby population in the river Vigda (N_e_ = 145), despite the annual number of sea trout entering the respective catchments being approximately equal. These N_e_ estimates are subject to uncertainty, as are the numbers of spawners entering the rivers, and should therefore be regarded as approximations. Moreover, the number of sea trout entering the Fremstad catchment is not directly associated with the presented N_e_ estimates. Also, the ecological conditions are different in the two populations, which means that the assumptions underlying the N_e_ estimates may be violated in different ways and with different outcomes on the estimates. For example, the N_e_ estimate for Vigda is probably inflated by migration (Waples, 2006), as Vigda's low pairwise F_ST_ to nearby populations (Table S4) indicates a degree of gene flow. However, regardless of the challenges relating to estimates of effective population size, a considerable non‐migratory component among spawners in Fremstad is also supported by life‐history data from scale reading, which shows that 17% of trout caught in lakes Storvatnet and Litjvatnet were non‐migratory spawners.

Despite being a highly adaptive, widely introduced species, many sea trout populations are under threat in their native range (Almodóvar et al., 2012; Dauwalter et al., 2020; Fiske et al., 2024). Like other diadromous fishes, the viability of individual populations is impacted by conditions at sea and in freshwater, both of which are currently under pressure. A recent assessment of 1279 Norwegian sea trout populations, including the Fremstad population, found that ~40% of populations were severely impacted by anthropogenic effects. Increased mortality induced by elevated levels of salmon lice infestations from Atlantic salmon aquaculture was identified as the most severe threat, and for the sea trout population in Fremstad, salmon lice was found to be the major threat (Fiske et al., 2024). This marine parasite may directly alter the evolutionary trajectory of anadromy in brown trout (Bolstad et al., 2025), as anadromy will likely be selected against under conditions where salmon lice reduce the survival and reproductive success of anadromous individuals (Thorstad et al., 2015). A fitness‐based indicator for the effect of salmon lice spilling back from farmed salmonids to wild sea trout has recently been developed (Bolstad et al., 2025) and may be included in the Norwegian ‘traffic‐light’ system (Holstad et al., 2025; Næsje et al., 2025), which regulates the biomass of farmed salmonids in net pens along the Norwegian coast (Stige et al., 2024). Knowledge of the anadromous and resident components of spawners in the Fremstad catchment and detailed monitoring of movement in and out of the catchment (Paterson et al., 2021) can be used together with information on local salmon lice abundance (Myksvoll et al., 2018; Nilsen et al., 2024; Sandvik et al., 2016) to empirically test and improve the model presented in Bolstad et al. (2025).

Riverine ecosystems are highly threatened on a global scale (Reid et al., 2019), and it is estimated that European rivers are fragmented by at least 1.2 million barriers that may restrict the movement of freshwater organisms (Belletti et al., 2020). In central Norway, around 90% of sea trout spawning habitat has been lost due to poorly designed culverts, canalization and degradation of small streams (Bergan, 2013; Bergan & Nøst, 2017; Hol, 2018). The Fremstad catchment hosts a genetically divergent sea trout population, with an effective population size large enough to preserve its level of genetic variation and quantitative characters over time (Lynch & Lande, 1998). Trout in the Fremstad catchment, both the anadromous and resident components, depend on available stream habitat for reproduction. Each of the seven spawning streams is small (anadromous areas of 100–2000 m^2^), and some are impacted by canalization and agricultural runoff. This study shows how a few small streams in a watercourse can support a large and stable trout population, thus highlighting the importance of conserving a variety of habitat types to safeguard trout populations into the future.

## AUTHOR CONTRIBUTIONS

Tor F. Næsje, Ingerid J. Hagen, Sten Karlsson and Rachel A. Paterson designed the research. Sten Karlsson and Ingerid J. Hagen compiled the genetic data, and Ingerid J. Hagen led the analyses. Geir H. Bolstad, Ola H. Diserud and Ola Ugedal contributed to statistical analysis. Ingerid J. Hagen wrote the paper with input from all authors. Tor F. Næsje initiated the project.

## FUNDING INFORMATION

This project was funded by the Norwegian Institute for Nature Research and the Norwegian Environment Agency.

## Supporting information


**Table S1.** Summary of sampling locations, the number of fish caught at each site and those that were used in downstream analyses, reach length, sampling year, age of individuals and sample type. Age 0 is young of the year (0+).
**Table S2.** The effective number of parents (N_eB_) for each brood year with the respective 95% confidence intervals and the number of full sibs removed in each spawning stream.
**Table S3.** Pairwise FST values (below diagonal) for groups of individuals collected in the spawning streams during the years 2022 and 2023 (−22 and −23), and different reaches within the streams (R1 and R2). Above the diagonal are 95% confidence intervals for the respective FST values. The different colours indicate pairwise values within the respective streams.
**Table S4.** Pairwise F_ST_ values (below diagonal) for Fremstad and 15 other regional populations. Above the diagonal are 95% confidence intervals for the respective F_ST_ values.
**Figure S1.** Size distribution of juvenile brown trout collected in the spawning streams during June 2022 (upper left panel) and November 2023 (upper right panel). Because field work was conducted in early winter in 2023, juveniles collected in 2023 were larger than those collected in summer 2022. The lower left panel shows the size distribution of PIT market out‐migrating sea trout, and the lower right panel shows the size distribution of individuals caught in the lakes.
**Figure S2.** BIC values for clusters 1–13 from DAPC analysis.

## Data Availability

All data underlying this study have been deposited in Zenodo (accession 10.5281/zenodo.17405949).
