# Supplementary material for: Genetic composition of facultatively anadromous brown trout Salmo trutta L. in a freshwater‐lake system
Source: J Fish Biol. 2026 Jun 30;109(3):1747–59. doi: 10.1111/jfb.70553 (PMC13602100; doi:10.1111/jfb.70553)
Supplement: Supplementary file 1 [file JFB-109-1747-s001.docx]

# Supplementary Files for

Genetic composition of facultatively anadromous brown trout (*Salmo trutta* L.) in a freshwater-lake system

Ingerid J. Hagen^1*^, Rachel A. Paterson^1^, Sten Karlsson^1^, Ola H. Diserud^1^, Geir H. Bolstad^1^, Eva M. Ulvan^1^, Ola Ugedal^1^ & Tor F. Næsje^1^

1) Norwegian Institute for Nature Research, PO Box 5685 Torgarden, 7485 Trondheim, Norway

**Corresponding author:** Ingerid J. Hagen, Norwegian Institute for Nature Research, PO Box 5685 Torgarden, 7485 Trondheim, Norway. Email address: ingerid.hagen@nina.no

**Table S1.** Summary of sampling locations, the number of fish caught at each site and that were used in downstream analyses, reach length, sampling year, age of individuals and sample type. Age 0 is young of the year (0+)

| Habitat type | Location | Reach number | Reach length (m) | Year | N fish | Mean age (min-max yrs) | Sample type |
| --- | --- | --- | --- | --- | --- | --- | --- |
| Spawning stream | Bjørdalsbekken | - | 62  98 | 2022  2023 | 77 | 0.47 (0-1) | fin |
|  | Vollaelva | 1  2  1  2 | 123  102  144  89 | 2022  2022  2023  2023 | 169 | 0.69 (0-2) | fin |
|  | Rivdalsbekken | - | 452  271 | 2022  2023 | 144 | 0.65 (0-3) | fin |
|  | Musdalselva | 1  2  1  2 | 110  66  156  55 | 2022  2022  2023  2023 | 162 | 0.76 (0-3) | fin |
|  | Djupdalsbekken | - | 92 | 2022 | 55 | 0.60 (0-2) | fin |
|  | Stordalsbekken | - | 146  182 | 2022  2023 | 106 | 0.42 (0-1) | fin |
|  | Vassbekken | - | 159  314 | 2022  2023 | 112 | 0.15 (0-2) | fin |
| Lake | Storvatnet | - | - | 2021 | 190 | - | fin |
| Lake | Litjvatnet | - | - | 2021 | 95 | - | fin |
| River | Fremstadelva* |  | - | 2017, 2020, 2021 | 366 | - | PIT tag needles |
| River | Heggeelva** | - | - | 2023 | 180 | - | scale |

* Out-migrating sea trout captured in Fremstadelva at the outlet of Storvatnet, with status as sea trout confirmed from detection at the Heggaelva PIT tag antenna (Figure 1). ** Up-migratory sea trout captured in the trap at Heggeelva as they entred the Fremstad catchment from the sea.

**Table S2.** The effective number of parents (N_eB_) for each brood year with the respective 95% confidence intervals and the number of full sibs removed in each spawning stream.

| Location | Brood  year | N_eB_ | 95% CI | N full sibs  removed |
| --- | --- | --- | --- | --- |
| Bjørdalsbekken | 2020  2022 | 57  85 | 37-98  51-159 | None  1 |
| Vollaelva | 2020  2021  2022 | 60  54  70 | 43-89  31-113  45-125 | 14  1  2 |
| Rivdalsbekken | 2020  2022 | 23  68 | 14-43  47-103 | 35  5 |
| Musdalselva | 2020  2021  2022 | 58  95  64 | 40-89  49-366  41-100 | 5  None  4 |
| Djupdalsbekken | 2020  2021 | 24  58 | 14-45  33-133 | 13  3 |
| Stordalsbekken | 2020  2022 | 50  73 | 30-94  49-110 | 5  4 |
| Vassbekken | 2021  2022 | 26  81 | 15-46  56-117 | 15  8 |

**Table S3:** Pairwise F_ST_ values (below diagonal) for groups of individuals collected in the spawning streams during the years 2022 and 2023 (-22 and -23), and different reaches within the streams (R1 and R2). Above the diagonal are 95% confidence intervals for the respective F_ST_ values. The different colors indicate pairwise values within the respective streams.

**Table S4:** Pairwise F_ST_ values (below diagonal) for Fremstad and 15 other regional populations. Above the diagonal are 95% confidence intervals for the respective F_ST_ values.

**Figure S1:** Size distribution of juvenile brown trout collected in the spawning streams during June 2022 (upper left panel) and November 2023 (upper right panel). Because field work was conducted in early winter in 2023, juveniles collected in 2023 were of a larger size than the ones collected in summer 2022. The lower left panel shows size distribution of PIT market out-migrating sea trout, and the lower right panel shows the size distribution of individuals caught in the lakes.

**Figure S2:** BIC values for clusters 1-13 from DAPC analysis.

**Figure S3:** Eigenvalues from PcoA in Figure 2, where the first two PC are plotted.
